# Large brains, short life: selection on brain size impacts intrinsic lifespan

**DOI:** 10.1098/rsbl.2019.0137

**Published:** 2019-05-15

**Authors:** Alexander Kotrschal, Alberto Corral-Lopez, Niclas Kolm

**Affiliations:** Department of Zoology/Ethology, Stockholm University, Stockholm, Sweden

**Keywords:** brain size, ageing, lifespan

## Abstract

The relationship between brain size and ageing is a paradox. The cognitive benefits of large brains should protect from extrinsic mortality and thus indirectly select for slower ageing. However, the substantial energetic cost of neural tissue may also impact the energetic budget of large-brained organisms, causing less investment in somatic maintenance and thereby faster ageing. While the positive association between brain size and survival in the wild is well established, no studies exist on the direct effects of brain size on ageing. Here we test how brain size influences intrinsic ageing in guppy (*Poecilia reticulata*) brain size selection lines with 12% difference in relative brain size. Measuring survival under benign conditions, we find that large-brained animals live 22% shorter than small-brained animals and the effect is similar in both males and females. Our results suggest a trade-off between investment into brain size and somatic maintenance. This implies that the link between brain size and ageing is contingent on the mechanism of mortality, and selection for positive correlations between brain size and ageing should occur mainly under cognition-driven survival benefits from increased brain size. We show that accelerated ageing can be a cost of evolving a larger brain.

## 1. Introduction

A long life can be achieved by minimizing extrinsic causes of death and/or decelerating intrinsic ageing. To reach old age, animals therefore ideally need to age slowly and avoid becoming prey. Ageing often evolves as a consequence of extrinsic mortality, where high rates of extrinsic mortality are thought to lead to increased ageing [1]. A large brain may aid in decreasing extrinsic mortality. Indeed, large-brained animals generally live longer than short-lived ones [2]. One important reason for this is that larger-brained animals usually display greater cognitive abilities [3–5], which they can use to devise adequate behavioural responses to unpredictable predation pressures and foraging challenges [6]. Hence, large-brained animals survive better in novel situations [7,8]. As large brains can mediate a decrease in extrinsic mortality and experimentally changing extrinsic mortality often drives the evolution of ageing rates [9–11], evolving larger brains may be evolutionarily correlated with slower ageing. However, brain tissue is among the most energetically costly tissues per unit of mass [12]. This cost is often used to explain the negative relationships (trade-offs) between brain size and other traits such as gut size [13–15] or juvenile growth rate [16]. Evolving a larger brain could therefore divert energy away from somatic maintenance and lead to faster ageing. In this context, the ‘disposable soma theory’ suggests that organisms adjust their investment of energy and resources into either somatic maintenance or other costly traits such as reproduction [17]. Increasing investment into brain development and/or higher ‘running costs’ of larger brains could therefore lead to a lack of somatic maintenance and faster ageing. The present ambiguity over the mechanisms that affect the correlation between brain size and ageing means that further analysis is important to fully understand this link. In particular, this is the case for the question of how intrinsic ageing is affected by brain size under conditions limiting correlated selection, where no empirical data are presently available.

Here we use brain-size selected guppies (*Poecilia reticulata*) to test the effect of brain size on intrinsic ageing while removing the possibilities for behaviourally mediated brain size survival advantages. For this, we kept males and females of three large- and three small-brained selection lines, which differ by 12% in relative brain size [3], under near ad libitum food availability in individual tanks and determined their lifespan. If developing a larger brain diverts energy from somatic maintenance we expect small-brained animals to live longer. However, if there is a genetic correlation between evolving a larger brain and longer lifespan we expect large-brained animals to live longer.

## 2. Material and methods

### (a) The brain-size selected guppies

We examined the relationship between brain size and intrinsic longevity in lines of Trinidadian guppies that were artificially selected for large or small relative brain size [18]. These selection lines differ in relative brain size (corrected for body size) by 9% in F_2_ and up to 14% in F_3_ [3] and while the small-brained animals show faster juvenile growth [19], adult body size does not differ between the lines [16]. Likewise, although large-brained guppies have smaller guts [3], there is no difference in feeding propensity or assimilation efficiency between the lines under ad libitum feeding conditions [16]. Here we used 60 male and female F_4_ fish. All fish were removed from their parental tanks after birth, separated by sex at the first onset of sexual maturation (approx. six weeks of age) and then kept in individual 4 l tanks for the rest of their lives. The tanks contained 2 cm of gravel, java moss, snails (*Planorbis* sp.) and continuously aerated water. We allowed for visual contact between the tanks. The room temperature was 26°C with a 12 : 12 light : dark schedule. Fish were fed a diet of flake food and freshly hatched brine shrimp 6 days per week. We use the term ‘near ad libitum’ as during every feeding we provided slightly more food than every fish could finish within 5 min. Snails consumed all uneaten food during the night. Most casualties were registered during the standard feeding routine; additionally, we checked every tank on a weekly basis to confirm the presence of a live fish. Note that we did not investigate the cause of death, but we never observed any obvious signs of sickness or macro parasites. Sometimes we observed a decrease in body condition and activity in the days before we found a fish dead. Two fish jumped out of their holding tanks and had to be excluded from the dataset. All data can be found in the electronic supplementary material.

### (b) Statistical analyses

To determine whether selection for relative brain size influences intrinsic lifespan we assessed potential differences in survival time using a proportional hazards-based mixed-effects Cox regression model [3,16]. As the dependent variable, we used individual survival (was it present/absent) at every census; as fixed factors sex (males/females), brain size selection regime (small/large), and their interaction, and as random effects we included replicate nested in brain size (three replicates, each with one large- and one small-brained line). We removed the non-significant interaction (sex * selection: *p* = 0.56) from the final model. The analysis was done using the ‘coxme’ package in R [20].

## 3. Results

**Figure 1.**
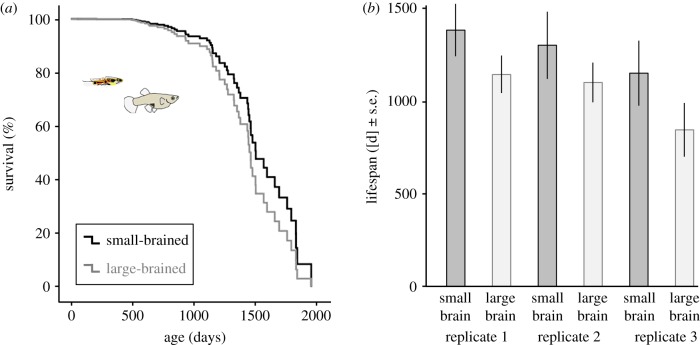
Brain-size selected guppies differ in lifespan. Large-brained animals die earlier than small-brained animals when kept individually and fed near ad libitum. Panel shows (*a*) survival curves controlled for replicate; (*b*) shows raw lifespan data for every selection line. Note that all three replicates show a similar trend of shorter lifespan in large- compared with small-brained animals. (Online version in colour.)

The mean lifespan for all experimental animals was 3 years, 1 month and 26 days. The last individual, a small-brained male, died over 5 years (1952 days) after it was born. We found that, while males and females did not differ in lifespan, small-brained animals survived on average 22% longer than large-brained animals (Cox regression: brain size selection regime: *z* = 2.78, *p* = 0.005, sex: *z* = 0.13, *p* = 0.900; table 1 and figure 1*a*). Note that figure 1*a* shows mean survival values controlled for replicate. Large-brained fish lived on average (±s.e.) for 1044 ± 68 days and small-brained fish for 1274 ± 95 days, and the brain size selection effect was visible in all three replicates, as illustrated by raw data shown in figure 1*b*.

**Table 1.** Results of a proportional hazards-based Cox regression model investigating the influence of sex and brain size selection on intrinsic lifespan of large- and small-brained guppies.

	coeff	Exp (coeff)	s.e. (coeff)	*z*	*p*-value
brain size selection	0.544	1.723	0.196	2.78	**0.005**
sex	0.025	1.025	0.195	0.13	0.900
*random effect*	*variance*				
replicate (selection)	0.0004				
replicate	0.0106				
individual	0.4809				

## 4. Discussion

Here we show that artificial selection for relative brain size leads to a shorter lifespan in large- compared with small-brained animals. Our results therefore provide no support for any genetic correlation between evolving a larger brain and slower ageing. Instead we find evidence for faster ageing in large-brained animals as they died earlier. Accelerated ageing can thus be added to the list of costs of increased encephalization.

Our experimental animals may have shifted allocation of metabolic resources between investment into brain size and somatic maintenance leading to faster ageing in the large-brained lines. In line with this suggestion, we previously found in these selection lines that large-brained animals have a lower innate immune response compared with small-brained animals [21]. Additional assays on these lines could in the future test for differences in, for instance, cellular repair mechanisms in response to oxidative damage. If there were a cost of a large brain in decreased somatic maintenance, we would expect signs of increased cellular oxidative damage [22] or decreased ability to counteract damage of reactive oxidants [23] in large-brained animals. Complimentarily, operating a larger brain is expected to be metabolically more expensive than operating a smaller brain [12]. A large brain may hence increase ageing via an increase in metabolic rate and again increased production of reactive oxidants (‘free radical theory of ageing’ [24]). However, large-brained guppies do not consume more food than small-brained guppies, their digestive efficiency is similar [16] and their physiological stress response is even decreased compared with small-brained guppies [25]. Assays of metabolic rate will be necessary to test if this mechanism indeed contributes to faster ageing in large-brained animals.

Sex differences in lifespan and ageing are widespread across the tree of life (reviewed in [26]). However, we found no difference in lifespan between males and females. This is unexpected, because female guppies typically survive much longer than male guppies [8,27,28]. This is usually attributed to the higher conspicuousness to predators in males due to their elaborate coloration. Such a sex-specific difference in survival pressure has been suggested to lead to sex-specific ageing rates where the sex with the higher extrinsic death risk evolves faster ageing [29]. That we did not find this effect may suggest that there is limited scope for evolution of sex-specific ageing rates, at least in this system.

Evolutionary patterns of life history are relatively consistent across vertebrate taxa. If this is the case for the brain-size/lifespan trade-off found in our guppies, we predict that this is relevant especially for larger and longer-lived species where ageing is often a major cause of death. In fact, autopsy data from humans show that older deceased individuals have smaller brains than people dying at younger age [30]. As age-dependent shrinkage in brain mass likely contributes to this pattern [31], long-term repeated brain mass measurements in the same individuals until death are required to clarify whether there in fact exists a brain-size/lifespan trade-off also in humans. Our results also imply that the link between brain size and ageing could be contingent on the mechanism of mortality. We propose that when survival benefits are driven by cognitive advantages from larger brain size, the correlation between brain size and ageing should be positive. However, when there is a lack of cognition-driven survival, the pattern should be reversed. These predictions can be tested by future studies.

## Supplementary Material

Data file Kotrschal et al. Brain size and lifespan

## Data accessibility

All data can be found in the electronic supplementary material to this manuscript.
